# USING INERTIAL MEASUREMENT UNITS (IMUS) TO DETECT MOBILITY DECLINES IN MIDDLE-AGED INDIVIDUALS

**DOI:** 10.1093/geroni/igad104.2183

**Published:** 2023-12-21

**Authors:** Elsa Davis, Mithran Periassamy, Braden Stock, Peter Altenburger, Satyajit Ambike, Jeffrey Haddad

**Affiliations:** Purdue University, West Lafayette, Indiana, United States; Purdue University, West Lafayette, Indiana, United States; Purdue University, West Lafayette, Indiana, United States; Indiana University, Indianapolis, Indiana, United States; Purdue University, West Lafayette, Indiana, United States; Purdue University, West Lafayette, Indiana, United States

## Abstract

Valid and reliable assessments of mobility are routinely used in clinics to track patient progress, assess the need for assistive devices, and predict risk of falling. These tests require patients to perform tasks that span varying degrees of difficulty so that therapists can observe changes in mobility and balance that can occur as a function of age and neurological disease. Evidence suggests that subtle gait changes which begin in middle-age can predict the future onset of mobility difficulties. However, standard clinical tests which rely on visual observation are designed to capture easily observable balance and gait problems in patients, rather than small changes that begin in middle-age. In this study we examined mobility changes that occur as a function of age using a common clinical test, the Functional Gait Assessment (FGA). Both middle aged (n=8) and young adult (n=12) participants completed the FGA while wearing small sensors, called inertial measurement units (IMUs). IMUs were placed on each ankle and the trunk to capture subtle differences in gait patterns. Participants also completed a health history questionnaire. Preliminary data suggest gait variability was increased in middle-aged adults (especially towards the end of the gait cycle), in the more difficult tasks. Subtle changes in gait velocity and leg acceleration during the swing phase of gait also appears to emerge by middle-age. Using IMUs to capture changes in gait patterns of middle-aged participants may help to identify those who are at future risk of having mobility difficulties and significant health risk.

